# O6-5 Can the development of a physical activity counseling network help fostering effective local policies for the attention of inactive people? Mugiment Gipuzkoa

**DOI:** 10.1093/eurpub/ckac094.045

**Published:** 2022-08-29

**Authors:** Iñaki Iturrioz, Unai Asurmendi, Jon Amiama

**Affiliations:** 1 Sport, Provincial Council of Gipuzkoa, San Sebastian, Spain; 2 Sport, BPXport Kirol Zerbitzuak SLU, San Sebastian, Spain

**Keywords:** physical activity, policy, Gipuzkoa, counseling, network

## Abstract

**Issue:**

In the province of Gipuzkoa, 34.1% of women and 32.1% of men do not reach the minimum levels of healthy physical activity. On the other hand, there is an important influence of social determinants, the gap on physical activity between the richest 10% and the poorest 10% is 28 points.

**Description:**

Mugiment Gipuzkoa is a multicomponent and multilevel initiative whose objective is the promotion of physical activity in the population. One of the main strategies is the development of a network of physical activity counseling services (PACS). In this network the autonomous, provincial and local government of different sectors such as health, sports, social services and education are called to act in coordination. The objective of the PACS network, is to cause the change of behavior in the inactive population, but also to reorient the policies of local entities regarding physical activity. The PACS connect sports services to the local services of different sectors, articulating referral protocols.

**Results:**

At the beginning of 2020 Gipuzkoa has 23 PACS in 16 municipalities. In 2018, 16 PACS corresponding to 12 municipalities have been evaluated. 778 people have attended to the services, the user profile is of an adult or aged woman, inactive, who came from the health center or autonomously. Most of them (73%), opted for autonomous physical activity. Pre-post methodology has been inplemented using a sample of 365 participants. Analized the clinical health history, we observe a positive trend on BMI, physical activity and less use of health resources at six months.

**Lessons:**

Coordinated action between different levels of the administration and different sectors is key to the accompaniment of local entities when developing policies aimed at the inactive population. In the case of social and educational services, the experience of collaboration is limited, so it is necessary to deepen these sectors.

**Main message:**

The establishment of a network of services for orientation to physical activity can serve to foster the development of effective local policies that focus on inactive people.

